# Antibacterial Activities of *Dodonaea viscosa* using Contact Bioautography Technique

**DOI:** 10.3390/molecules14031332

**Published:** 2009-03-26

**Authors:** Muhammad Khurram, Murad Ali Khan, Abdul Hameed, Naz Abbas, Abdul Qayum, Humaira Inayat

**Affiliations:** 1 Department of Microbiology, Quaid-i-Azam University, Islamabad, Pakistan; 2 Medicinal Botanical Centre, PCSIR Laboratories, University Road, Peshawar, Pakistan; 3 Department of Chemistry, Kohat University of Science and Technology, Kohat-26000 Pakistan; 4 Department of Pharmacology, Peshawar Medical College, Warsak road, Peshawar Pakistan; 5 Institute of Chemical Sciences, University of Peshawar, Peshawar Pakistan

**Keywords:** *Dodonaea viscosa*, Sapindaceae, Antibacterial, Contact bioautography, MIC, MBC.

## Abstract

The crude ethanolic extract and *n*-hexane, dichloromethane, ethyl acetate, *n*-butanol and aqueous fractions of *Dodonaea viscosa* were analyzed for antibacterial potential against four Gram positive bacteria: *Bacillus subtilis, Bacillus cereus, Micrococcus luteus, Staphylococcus aureus,* and three Gram negative bacteria: *Escherichia coli, Salmonella typhi, Pseudomonas aeruginosa*. Preliminary screening showed inhibition against *Staphylococcus aureus*, *Micrococcus luteus, Escherichia coli* and *Pseudomonas aeruginosa*. The thin layer chromatograms of the fractions were then subjected to contact bioautography, which showed inhibition zone at different *R_f_* values against *Bacillus subtilis, Micrococcus luteus, Escherichia coli, Salmonella typhi and Pseudomonas aeruginosa,* indicating the presence of antibacterial components. The MIC of each fraction was determined through a 96-well micro-titer plate method. The non-viability of the organisms was ascertained by determining the MBC of the fractions.

## Introduction

The antibiotic era started in the 1950s, and from then onwards the use of plant antimicrobials declined [1]; although it was not the case as far as the traditional healing systems that heavily rely on the medicines from the natural sources, especially plants, are concerned. The emergence and spread of microbial resistance is growing each day, thereby necessitating the development of new antimicrobials of natural or synthetic origin [2]. As far as the natural sources are concerned, apart from the microbial sources, plants appear to be valuable antimicrobial resources. Plants can produce a large number of secondary metabolites that may exceed a hundred thousand molecules [3]; all of these don’t have antimicrobial potential, but some of them can produce significant activity against the human pathogens. This activity is not necessarily of the same magnitude as that of the prevailing antimicrobials, but still provides some scope for optimism [4]. Consequently, the search for such antimicrobials has intensified during recent times. This can be observed from the fact that whereas the number of articles published on the antimicrobial activities of medicinal plants during 1966 - 1994 was 115, the number of articles on the same subject appearing during 1995 - 2004, is 307 [5]; a more than two-fold increase in just one decade, showing the growth of interest in the search for antimicrobials of natural origin.

*Dodonaea viscosa* (L.) Jaeq. commonly known as Ghawraskay (Pushto), is a viscid shrub that grows to a height of 2 m. It is used in folk medicine as a remedy for fever, rheumatism and gout. The crude extract has inhibitory effects against *Staphylococcus aureus, Streptococcus pyogenes, and Corynebacterium dephtherieae*, but no activity against *Escherichia coli* and *Pseudomonas aeruginosa,* thereby suggesting potential against notable Gram positive organisms [6]. Coxsackie virus B3 and influenza A virus were inhibited, demonstrating the antiviral potential, while the yeast *Candida albicans,* conidiophore *Aspergillus fumigatus* and dermatophyte *Trichophyton rubrum* were not inhibited [6]. More recently the antifungal activity of *Dodonaea viscosa* against 40 *C. albicans* isolates was reported [7]. It has also shown anti-inflammatory effects in experimental animals [8].

Phytochemical investigations of *Dodonaea viscosa* have resulted in the isolation of flavonoids [9,10,13], saponins [11], and diterpenes [12]. Notable among these compounds are pinocembrin, santin, penduletin, alizarin, 5-hydroxy-3,6,7,4’-tetramethoxy flavone, 5,7,4’-trihydorxy-3,6-dimethoxy flavone, isorhamnetin-3-rhamnosylgalactoside, 5,7-dihydroxy-3’-(hydroxymethylbutyl)-3,6,4’-tri-methoxy flavones [9], 5,6,4’-trihydroxy-3,7-dimethoxy flavone [13], viscosol [10], hautriwaic acid [12,13], dehydrohautriwaic acid, methyl dodonates [12], *ent*-15,16-epoxy-3β,8α-dihydroxy-9αH-labda-13(16),14-diene[13], dodonoside A and dodonoside B [11]. This plant has not been thoroughly studied for antimicrobial activities, so in this study we have subjected the ethanolic extract and subsequent fractions of *Dodonaea viscosa* to assays to establish their antibacterial potential.

## Results and Discussion

Geitie *et al*. [6] reported the absence of activity against Gram negative organisms, but interestingly in our study (Table 1) we have observed promising antimicrobial activity against both Gram positive and negative organisms.

**Table 1:** Antibacterial activity of Crude and Aqueous Extracts of *Dodonaea viscosa.*

	Average Zone of Inhibition (in mm)			
***Bacteria***	**Crude extract**	**Aqueous extract**	**Positive Control**	**Negative Control**
*Staphylococcus aureus(G^+ve^)*	12.0 ± 0.3	0.0	41.0 ± 1.1†	0.0
*Micrococcus luteus (G^+ve^)*	12.9 ± 0.2	0.0	43.0 ± 0.5*	0.0
*Bacillus subtilis (G^+ve^)*	13.3 ± 0.2	0.0	40.0 ± 0.4†	0.0
*Bacillus cereus(G^+ve^)*	0.0	0.0	32.4 ± 0.4†	0.0
*Escherichia coli (G^-ve^)*	11.0 ± 0.2	0.0	30.0 ± 0.5*	0.0
*Pseudomonas aeruginosa (G^ve^)*	12.4 ± 0.4	0.0	32.0 ± 0.5†	0.0
*Salmonella typhi(G^-ve^)*	0.0	0.0	33.0 ± 0.3*	0.0

* Chloramphenicol = 1 mg/mL; † Ciprofloxacin = 1 mg/mL; G ^+ve^ = Gram Positive; G ^-ve^ = Gram Negative

**Figure 1:**
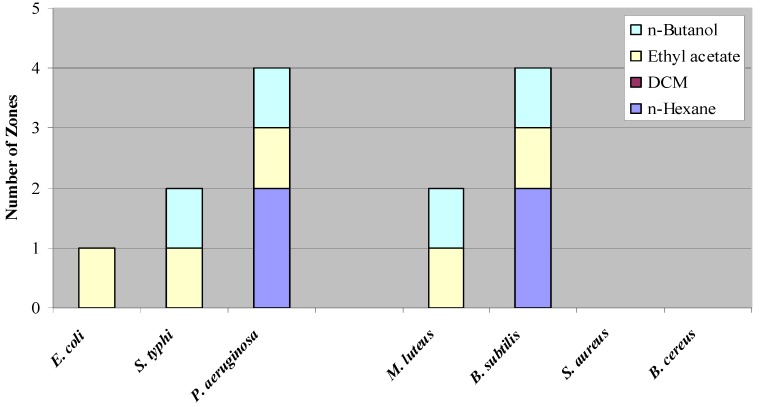
Number of Inhibition Zones Against Test Organisms.

Figure 1 shows the number of zones observed against the various test organisms. It is clear from the figure that Gram negative organisms are more sensitive than the Gram positive organisms. The degree of sensitivity and location of the zones with respect to related *R_f_* value are listed in Table (2). This inhibitory potential towards the Gram negative organisms was later confirmed when the crude extracts were subjected to contact bioautography. The presence of multiple inhibition zones against these organisms as well as the *S. typhi* indicates the presence of entities capable of suppressing their growth. 

Figure 1 shows the number of zones observed against the various test organisms. It is clear from the figure that Gram negative organisms are more sensitive than the Gram positive organisms. The degree of sensitivity and location of the zones with respect to related *R_f_* value are listed in Table (2). This inhibitory potential towards the Gram negative organisms was later confirmed when the crude extracts were subjected to contact bioautography. The presence of multiple inhibition zones against these organisms as well as the *S. typhi* indicates the presence of entities capable of suppressing their growth. 

**Table 2:** Location and Prominence of Zones of Inhibition at Different *R_f_* values of *Dodonaea viscosa* fractions against Test Bacteria.

	*Zone/(s) of Inhibition at R_f_ value of Fraction*			
Bacteria	*n*-Hexane	DCM	Ethyl acetate	*n*-Butanol
*Staphylococcus aureus*	-*	-	-	-
*Micrococcus luteus*	-	-	0.06	0.03
			(++)	(++)
*Bacillus subtilis*	0.14	-	0.03	0.06
	0.42			
	(++)		(++)	(++)
*Bacillus cereus*	-	-	-	-
*Escherichia coli*	-	-	0.03	-
			(+)	
*Salmonella typhi*	-	-	0.06	0.04
			(+)	(+)
*Pseudomonas aeruginosa*	0.14	-	0.06	0.04
	0.54			
	(++)		(++)	(++)

Degree of Inhibition: ++ = Prominent; + = Moderate; - = Nil;

* No Zone of Inhibition at any point of the TLC plate

The results shows good activity against *B. subtilis*, with prominent inhibition zones for the *n*-hexane (two zones of inhibition), ethyl acetate (one zone of inhibition) and *n*-butanol (one zone of inhibition) extracts. *M. luteus* has one zone of inhibition each for the ethyl acetate and *n*-butanol fractions. Interestingly, *S. aureus*, that gave a 12.0 ± 0.3 mm zone of inhibition (Table 1) failed to produce any zone of inhibition in the contact bioautography, which is most probably due to the synergestic action between the compounds present in the crude extract that got fractionated and later separated through the TLC, a reduced level or some chemical alteration of the active compound, thereby, resulting in the loss of anti-staphylococcal action.

The results given in Table 2 shows that ethyl acetate fraction was active against five out of seven tested organisms, followed by the *n*-butanol fraction that inhibits four organisms and the *n*-hexane fraction that is inhibitory to two organisms. An interesting observation with respect to the *n*-hexane fraction is the presence of multiple zones of inhibition, i.e., two zones each against *B. subtilis* (at *R_f_* =0.14 and 0.42) and *P. aeruginosa* (at *R_f_* =0.14 and 0.54). The antibacterial spectrum against Gram positive and Gram negative organisms, thereby, suggests presence of antibacterial entities that are capable of targeting either the bacterial wall or intracellular targets like bacterial ribosomes, etc.

**Figure 2:**
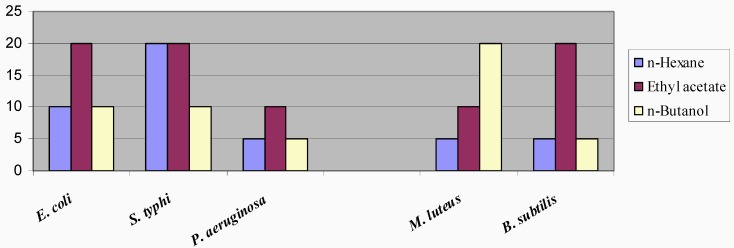
Minimum Inhibitory Concentrations (mg/ml) of Different Fractions of *Dodonaea viscosa* against Test Organisms.

Minimum inhibitory concentrations (MICs) of the crude fractions (Figure 2) were found to be within the range of 5 – 20.0 mg/mL. The high levels of the MIC's of the fractions are attributable to the facts that the active components are present in low concentrations, or there are some antagonistic components present that serve as growth promoters for the bacteria, thereby, necessitating the presence of high amount of the fraction to inhibit the growth.

The minimum bactericidal concentration (MBC) data (Table 3) shows an extension of the MIC data. It depicts the concentrations of the fractions that completely eliminated (99.9% reduction) the viable bacteria under test. On the basis of the data it can be concluded that *n*-hexane fraction has a higher bactericidal potential as compared to the ethyl acetate and *n*-butanol fractions.

**Table 3:** Minimum Bactericidal Concentrations (mg/mL) of Different Fractions of *Dodonaea viscosa.*

*Bacteria*	Positive Control	*n*-hexane	ethyl acetate	*n*-butanol
***Gram Positive***				
*Micrococcus luteus*	Chloramphenicol	10	20	>20
*Bacillus subtilis*	Ciprofloxacin	10	20	10
***Gram Negative***				
*Escherichia coli*	Chloramphenicol	20	>20	10
*Salmonella typhi*	Chloramphenicol	>20	>20	20
*Pseudomonas* *aeruginosa*	Ciprofloxacin	10	20	10
			

*P. aeruginosa* is an environmental inhabitant that is also an opportunistic pathogen. Its virulence is due to the production of exotoxin A, endotoxins, several proteolytic enzymes, alginates, pili, and hemolysins. It has been found to be involved in respiratory tract, urinary tract, bloodstream, and central nervous system infections of nosocomial origin [16]. With growing resistance against the prevailing antibacterial agents, observations are that this pathogen is becoming resistant against gentamicin, ciprofloxacin [17], tetracycline, chloramphenicol, and norfloxacin [18]. *E. coli* is involved in causing severe infections of the urinary tract (of both community and nosocomial origin), sepsis, meningitis, and *E. coli*-associated diarrheal diseases (Enteropathogenic *E. coli*, Enterotoxigenic *E. coli*, Enteroheamorrhagic *E. coli*, Enteroinvasive *E. coli*, and Enteroaggregative *E. coli*) [19]. *E. coli* resistance against fluoroquinolones, penicillins, cephalosporins, aminoglycosides [20] and sulfamethoxazole are reported [21]. *S. typhi* the causative agent for the enteric fevers, enterocolitis, and septicemia [22] is also becoming resistant against the antibacterial like ampicillin, tetracycline and chloramphenicol [23].

It is now an accepted fact that there is an increased resistance against the prevailing antimicrobials and our armory against these microorganisms is running out at a very quick pace. In order to withstand this challenge we need to develop new antimicrobials, which in itself is a great challenge and requires a huge investment. Turning back towards the molecules from natural sources like plants can be a good option. If these molecules are not as potent as the conventional antimicrobials they can be used as adjuncts, enhancing their therapeutic effectiveness. The isolation of active principles can be facilitated by bioassay guided fractionation. The present study indicates the presence of molecules that could have some therapeutic value if isolated and studied further.

## Experimental

### Plant material

*Dodonaea viscosa* (L.) Jaeq. belongs to the Sapindaceae family. The aerial parts of the plant were collected from Kohat, NWFP (Pakistan) in September 2007. A specimen was matched with the reference voucher number 592, preserved in the Herbarium of Pakistan, Department of Plant Sciences, Quaid-i-Azam University, Islamabad, Pakistan 

### Extraction

The shade dried plant material was chopped into small pieces and ground to a fine powder. Ethanol (80% v/v) was used to soak the pulverized material in a percolator at room temperature. Three portions of the percolate were collected at an interval of 96 hours each. The portions were combined to obtain eight liters of crude extract.

### Fractionation

The crude ethanolic extract was sequentially partitioned with *n*-hexane (3 x 1,000 mL), dichloromethane (3 x 1,000 mL), ethyl acetate (3 x 1,000 mL), and *n*-butanol (3 x 1,000 mL). A rotary evaporator was used to concentrate the portions that yielded a gummy n-hexane (75 g), syrupy dichloromethane (70 g) and ethyl acetate (92 g), gummy *n*-butanol (81 g) and an aqueous fraction (126 g), respectively.

### Preliminary screening for the presence of antimicrobial activity

The crude extract was dissolved in 10% (v/v) dimethyl sulfoxide (DMSO, Fluka, Spain) in normal saline (0.9% w/v) to make the concentration equal to 3.2 mg/mL. The bacterial strains were cultured on nutrient agar (Merck, Germany) in 9 cm diameter plates, a single isolated colony of each test bacteria was picked and transferred to Nutrient Broth (Oxoid, UK) and incubated for a period of 18 hours except for the *Salmonella typhi* (4 hours incubation) and *Micrococcus luteus* (48 hours incubation) in a shaker. After incubation period the turbidity of the solutions were adjusted to 0.5 McFarland turbidity standard, using sterile nutrient broth. Hundred microliter of the inoculum was evenly spread over the entire area of 9 cm diameter Petri plates containing Muller Hinton Agar [Oxoid, UK] media, with the help of a sterile glass spreader and allowed to dry. With the help of a sterile cork borer, wells measuring 7 mm in diameter were made. Seventy microliter of crude extract solution was transferred aseptically into the wells. 10% (v/v) DMSO in normal saline and standard antibiotics served as negative and positive controls respectively. The plates were placed in upright position in refrigerator for a period of two hours in order to allow the materials to diffuse around the well area. The zones of inhibition were measured after an incubation of 24 hours at 37^o^C, in case of all the organisms, except for *Micrococcus luteus*, which was incubated for a period of 48 hours, in an upright position. All the tests were run in triplicate.

### Thin layer chromatography and Contact bioautography

The fractions were dissolved in methanol (1 mg/mL) and applied, with the help of a fine bore glass capillary tube, a volume of 50 µL of each fraction on the entire length of pre-coated glass TLC plates silica gel 60 F_254 _layer thickness 0.25mm (Kieselgel 60 F_254_, Merck, Germany) of size 20 x 20 cm. Various solvent systems were tried to get good separation and finally the solvent systems listed in Table 4 gave best separation, and therefore, were applied for the respective fraction. The developed chromatograms (TLC plates) were cut into pieces of 2 x 10 cm size with the help of a glass cutter, wrapped twice and sealed in polythene bags. They were then gas sterilized for a period of 18 hours using an ethylene oxide sterilizer (Axis, Turkey). 

**Table 4:** Solvent Systems for *Dodonaea viscosa* fractions.

*Fraction*	Solvent System
*n-Hexane*	CH_3_OH: CHCl_3_ (3:200)
Dichloromethane*	i). CH_3_OH: CHCl_3_ (1:20); ii). CH_3_OH: CHCl_3_ (3:10)
*Ethyl acetate*	*n*-Butanol: acetic acid: water (12:3:5)
*n-Butanol*	*n*-Butanol: acetic acid: water (12:3:5)

* TLC plates loaded with the dichloromethane fraction were first developed to a maximum with system (i) and then half developed with system (ii)

The bacterial strains were prepared, as mentioned above, in the nutrient broth. One hundred microliters of each of the inoculum was evenly spread, with the help of a sterile glass spreader. The seeded plates were allowed to dry under laminar flow. The pieces of TLC plates were then placed aseptically upon the bacterial lawn and were left for a period of two hours, in order to allow the materials from them to diffuse on to the seeded plates. Thereafter, TLC plates were removed from the surface with the help of sterile forceps and the plates were incubated in inverted position for 24 hours, in case of all the organisms except for *M. luteus*, which was incubated for 48 hours. The areas of inhibition were marked and relevant *R_f_* values were recorded. All the tests were run in triplicate.

### Minimum inhibitory concentration

The fractions that showed antibacterial potential were further assessed for the minimum inhibitory concentration (MIC), which is the minimal concentration of plant extract, or fraction thereof that inhibits the bacterial growth [14]. The stock solutions of the fractions were prepared in pure DMSO in strength of 160 g/L and stored at +4 °C in a refrigerator till their use. Hundred microliter of stock solution containing approximately 16mg of each fraction was transferred aseptically to the micro-titer plate and sequentially half diluted in sterile solution of DMSO in normal saline (9% v/v) such that the last well contained ≈32μg/ml. The bacterial strains were prepared using Muller Hinton Broth (Oxoid, UK) and hundred microliter of the adjusted inoculum (turbidity adjusted equivalent to 0.5 MacFarland) was transferred aseptically to the 96-well micro-titer plate (TPP, Switzerland) in all of the wells containing the test dilutions, negative control (DMSO in normal saline) and standard antibiotics (either Ciprofloxacin or Chloramphenicol) containing wells that served as positive controls. The micro-titer plates were incubated at 37^o^C for 24 hours for all the organisms except for the *M. luteus* that required an incubation period of 48 hours. The tests were run in triplicate. 

### Minimum bactericidal concentration

It is defined as the concentration of the antimicrobial that results in a 99.9% reduction in CFU/ml compared with the organism concentration in the original inoculum [15]. The micro-titer plates were prepared in the same manner as discussed above for MIC studies. After 24 hours incubation or 48 hours incubation in case of *M. luteus*, from each of the visibly clear wells containing plant test materials, negative control and positive control, hundred microliter samples were taken and spread evenly on the entire surface of nutrient agar plates with the help of sterile glass spreader. The plates were allowed to dry and then kept in inverted position in incubator at 37 ^o^C for 24 hours for all of the test organisms except the *M. luteus* that was incubated for 48 hours at 37 ^o^C. After the incubation period, the colonies were counted and compared with negative control. The concentration of the plant extract that completely inhibited the growth of the test organism, thereby, resulting in no growth or a 99.9% reduction in CFU/ml was taken as the MBC. The tests were run in triplicate.
